# First person – Phillipe O'Brien, Kai Guo, Stephanie Eid, Amy Rumora and Lucy Hinder

**DOI:** 10.1242/dmm.043836

**Published:** 2020-01-24

## Abstract

First Person is a series of interviews with the first authors of a selection of papers published in Disease Models & Mechanisms (DMM), helping early-career researchers promote themselves alongside their papers. Phillipe O'Brien, Kai Guo, Stephanie Eid, Amy Rumora and Lucy Hinder are first authors on ‘Integrated lipidomic and transcriptomic analyses identify altered nerve triglycerides in mouse models of prediabetes and type 2 diabetes’, published in DMM. Phillipe, Stephanie, Amy and Lucy are all postdoctoral researchers in the lab of Eva L. Feldman at the University of Michigan, MI, USA, investigating the pathogenesis of peripheral neuropathy using mouse models of obesity and diabetes. Kai is a postdoctoral researcher in the lab of Junguk Hur at the University of North Dakota, Grand Forks, ND, USA, and uses high-throughput genomic data analysis to understand the development of neurological disorders.


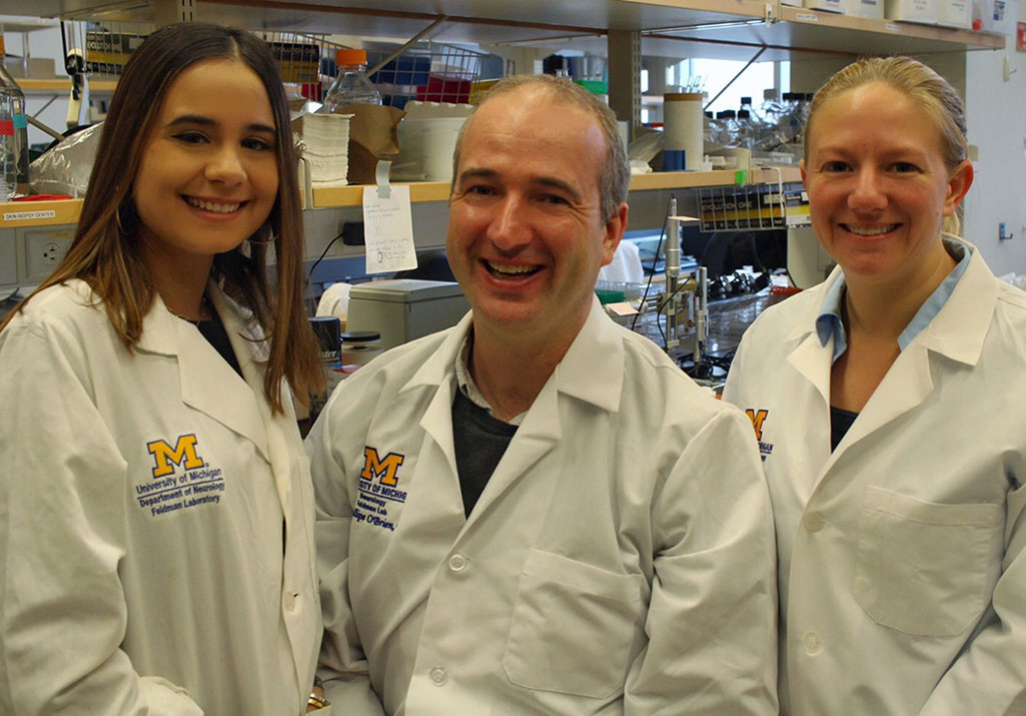


**Left to right: Stephanie Eid, Phillipe O'Brien and Amy Rumora**

**How would you explain the main findings of your paper to non-scientific family and friends?**

Obesity and diabetes are associated with the development of peripheral nerve damage, termed neuropathy. It is accepted that a diet rich in fat leads to nerve dysfunction in mouse models of obesity and diabetes. We have shown that switching the diet in mice from a high-fat to a normal diet, a form of lifestyle intervention, improves metabolic health and nerve function. Using this system of dietary reversal, our objective in this study was to explore the contribution of lipids to nerve damage. We found that levels of a specific class of lipids, triglycerides, and a protein involved in triglyceride synthesis, DGAT2, were elevated in the nerves of mice fed a high-fat diet but decreased in mice with dietary reversal. This study highlights that abnormal nerve lipid levels, as well as the type of lipids, contribute to nerve injury in obesity and diabetes.

“This study highlights that abnormal nerve lipid levels, as well as the type of lipids, contribute to nerve injury in obesity and diabetes.”

**What are the potential implications of these results for your field of research?**

Peripheral neuropathy is a common complication of obesity and type 2 diabetes that substantially impacts the patient's quality of life. Currently, there is no effective treatment and management. In patients with prediabetes and diabetes – common causes of peripheral neuropathy – management consists of tight glycemic control, which does not prevent peripheral neuropathy development. Thus, identifying the drivers of disease is important to develop therapeutic strategies. In this study, we demonstrate that nerve lipids, particularly triglycerides, are implicated in peripheral neuropathy, reinforcing the hypothesis that lipids are important factors in the onset and progression of neuropathy. Therefore, current research efforts should focus on identifying the mechanisms by which dietary lipids lead to nerve dysfunction.

**What are the main advantages and drawbacks of the model system you have used as it relates to the disease you are investigating?**

One of the main advantages of the high-fat-diet-fed mouse model is that, unlike the genetically modified *db/db* and *ob/ob* mouse models, these mice develop a gradual onset of disease, while retaining functional leptin signaling. Importantly, they consistently develop a robust peripheral neuropathy that mimics the human condition.

Another advantage of using this mouse model is that disease onset is inducible rather than spontaneous, which allows investigators to study disease onset and progression at different ages and stages.

A limitation of the high-fat-diet mouse model is that there is no consensus among research teams on the protocol for generating this model. These include: genetic background, strain, vendors of mice and diet, animal age, duration of high-fat feeding, percentage of fat, and diet composition. For this reason, agreeing on a common protocol is important to move the research forward.

“Another advantage of using this mouse model is that disease onset is inducible rather than spontaneous, which allows investigators to study disease onset and progression at different ages and stages.”

**What has surprised you the most while conducting your research?**

Although there is no pharmacological intervention for peripheral neuropathy, our findings once again show that lifestyle intervention through dietary reversal can improve nerve function in obesity and type 2 diabetes. Our results also highlight the role of nerve triglycerides in neuropathy, which can be reversed via dietary intervention.

**Describe what you think is the most significant challenge impacting your research at this time and how will this be addressed over the next 10 years?**

Type 2 diabetes and peripheral neuropathy are complex and multifactorial disorders, with both genetics and environment contributing to disease pathogenesis. Although the high-fat-fed mouse model serves as a robust animal model that recapitulates the human condition, the most significant challenge is establishing a unified approach across research groups. Ideally, over the next 10 years, a framework should be set into place with clearly defined protocols to overcome the current limitations.

“The lack of NIH funding opportunities is a major challenge faced by international postdoctoral fellows, which hinders career advancement.”

**What changes do you think could improve the professional lives of early-career scientists?**

The lack of NIH funding opportunities is a major challenge faced by international postdoctoral fellows, which hinders career advancement. Additionally, improved training opportunities in grant writing would be beneficial for early-career investigators. A standardized framework for training in grant writing would be helpful to ensure that all postdoctoral fellows have the resources they need to launch an independent scientific career.

**What's next for you?**

In the current study, we found that triglycerides and DGAT2, an enzyme that catalyzes the final step in triglyceride synthesis, are implicated in peripheral neuropathy. The next step would be to investigate the factors that regulate nerve triglycerides, with major emphasis on the role of DGAT2. These studies will confirm whether targeting triglycerides, or triglyceride synthesis pathways, will provide therapeutic benefit against peripheral neuropathy.
